# Venom Peptides as a Rich Source of Ca_v_2.2 Channel Blockers

**DOI:** 10.3390/toxins5020286

**Published:** 2013-02-04

**Authors:** Silmara R. Sousa, Irina Vetter, Richard J. Lewis

**Affiliations:** Institute for Molecular Bioscience, The University of Queensland, St Lucia, Queensland, 4072, Australia; E-Mails: s.rodriguesdesousa@uq.edu.au (S.R.S.); i.vetter@imb.uq.edu.au (I.V.)

**Keywords:** Ca_v_2.2, voltage-gated calcium channels, nociception, neurotransmitter, ω-conotoxins, venom peptides

## Abstract

Ca_v_2.2 is a calcium channel subtype localized at nerve terminals, including nociceptive fibers, where it initiates neurotransmitter release. Ca_v_2.2 is an important contributor to synaptic transmission in ascending pain pathways, and is up-regulated in the spinal cord in chronic pain states along with the auxiliary *α2δ*1 subunit. It is therefore not surprising that toxins that inhibit Ca_v_2.2 are analgesic. Venomous animals, such as cone snails, spiders, snakes, assassin bugs, centipedes and scorpions are rich sources of remarkably potent and selective Ca_v_2.2 inhibitors. However, side effects in humans currently limit their clinical use. Here we review Ca_v_2.2 inhibitors from venoms and their potential as drug leads.

## 1. Introduction

A wide diversity of venomous animals has evolved a large range of peptide toxins that target ion channels expressed in the neuronal and neuromuscular systems of prey and predators as part of efficient prey immobilization and deterrent strategies. Accordingly, many of the most selective ion channel modulators known originate from venoms (reviewed by [1]). These peptide toxins have evolved from a relatively small number of structural frameworks that are particularly well suited to address crucial issues such as, potency and stability [1]. While venoms from some spiders, such as *Phoneutria nigriventer*, are dominated by Na_v_ inhibitors [2]; in general, Ca_v_ inhibition dominates the pharmacology of spider venom peptides (reviewed by [3]). However, activity of many spider toxins at Ca_v_2.2 has not been characterized extensively, and many of these peptides preferentially target Ca_v_ channels other than Ca_v_2.2, such as Ca_v_2.1, Ca_v_2.3 or invertebrate Ca_v_ [4,5,6,7]. 

In contrast, a range of disulfide rich peptides from cone snails (conotoxins) preferentially inhibit Ca_v_2.2 (see Table 2; reviewed by: [8,9]). GVIA from *Conus geographus* has been used for many years as probe to discriminate Ca_v_2.2 from other closely related Ca_v_ channel subtypes ([10,11,12,13], for review see: [14]). In addition, several cone snail toxins have direct diagnostic and therapeutic potential [8,13,15,16] (reviewed by: [9,17]). A synthetic version of a Ca_v_2.2 channel blocker toxin ω-conotoxin MVIIA (ziconotide, Prialt^®^), from the venom of the cone snail *Conus magus* is currently in use clinically, validating Ca_v_2.2 as an analgesic target in humans [15,18]. Unfortunately, intrathecal administration and undesirable side effects have limited the clinical use of ziconitide [15,19]. Here we review Ca_v_2.2 channel inhibitor toxins from venoms, their pharmacological and structural properties as well as their therapeutic potential. 

## 2. Ca_v_ Channels

Calcium (Ca^2+^) currents in mammalian excitable cells have diverse pharmacological properties, and control essential physiological functions, including muscle contraction, hormone secretion, neurotransmitter release and nociceptive transmission. Voltage-gated calcium channels (Ca_v_), are multi-subunit complexes composed of different pore-forming/voltage-sensing α1 subunit types, and several α2δ, β and γ regulatory subunit isoforms. Genes encoding 10 pore-forming α1 (α_1A_–α_1I_ and α_1s_) as well as several splice variants have been identified and characterized. Ca_v_ superfamilies 1 and 2 require higher voltage steps to be activated, and are thus classified as high-threshold calcium channels, while superfamily 3 has a lower threshold for activation. High threshold currents include L-type (encoded by Ca_v_1.1–1.4 genes), N-type (Ca_v_2.2), P/Q-type (Ca_v_2.1) and R-type (Ca_v_2.3) channels, while T-type (Ca_v_3.1–3.3) calcium channels are low-threshold channels (Table 1) (for review see [14,20]).

**Figure 1:**
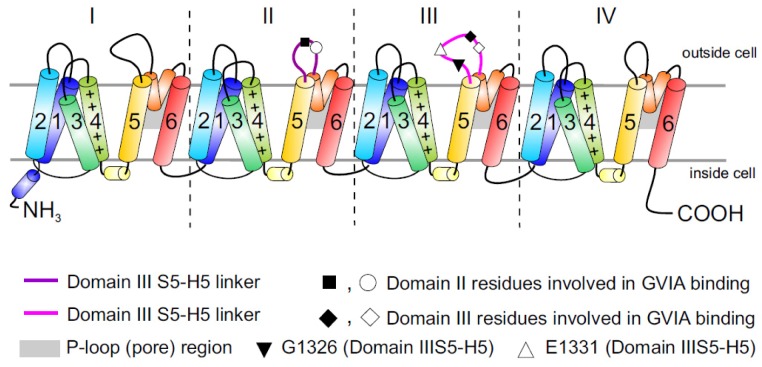
Topology of Ca_v_ channels: Represented is the pore-forming α1 subunit of the Ca_v_2.2 channels. This large protein consists of four homologous transmembrane domains (I–IV) and each domain contains six segments (S1–S6) and a membrane-associated P loop between S5 and S6 (represented in orange/grey) where the binding site of ω-conotoxins is localized. Circles, triangles and rectangles represent the localization of specific residues described to be important for binding of Ca_v_2.2 to the ω-conotoxin GVIA [11].

The primary structure of the Ca_v_ family has been determined by a combination of protein chemistry, cDNA cloning and sequencing [14,21,22]. Their hetero-oligomeric nature was established from biochemical glycosylation and hydrophobicity analyses. At least three auxiliary subunits, which regulate Ca_v_2.2 expression and function, have been defined. The ~190 kDa pore-forming transmembrane α1 subunit (~2000 amino acids), is organized in four homologous domains (I–IV), comprising six transmembrane α helices (S1–S6) and the pore-forming P loop between S5 and S6 (Figure 1) [14]. Studies on the structure and function of the related pore-forming subunits of Na^+^ and K^+^ channels have resulted in the identification of their principal functional domains [23]. The S4 segments form a key part of the voltage sensor module (Figure 1), moving outward and rotating under the influence of the electric field and initiating a conformational change that opens the pore. The external entrance to the ion conducting pore of the channel is lined by the P loop, which contains a pair of glutamate residues in each domain, required for Ca^2+^ ion selectivity. The inner pore is lined by the S6 segments (Figure 1), which forms the receptor site for the pore-blocking Ca_v_1 antagonist drugs (for review see: [22]). While no high-resolution crystal structure of the Ca_v_ is available to date, structures from related ion channels, in particular the recently determined bacterial voltage-gated sodium channel [24,25], promise to provide significant insight into the structure and function of Ca_v_ channels. 

**Table 1:** Physiological function and pharmacology of Ca_v_channel subtypes.

Ca_v _subtype	Current type	Localization	Antagonist class/Name	Physiological function
Ca_v_1.1	L	Skeletal muscle, transverse tubules	DHP, PHA, BTZ	Excitation-contraction coupling, gene regulation
Ca_v_1.2	L	Cardiac myocytes, smooth muscle myocytes, endocrine cells, neuronal cell bodies, proximal dendrites	DHP, PHA, BTZ	Excitation-contraction coupling, hormone secretion, gene regulation
Ca_v_1.3	L	Endocrine cells, neuronal cell bodies and dendrites, cardiac atrial myocytes and pacemarker cells, cochlear hair cells	DHP, PHA, BTZ	Hormone secretion, gene regulation, tonic transmitter release
Ca_v_1.4	L	Retinal rod and bipolar cells, spinal cord, adrenal gland, mast cells	DHP, PHA, BTZ	Tonic neurotransmitter release
Ca_v_2.1	P/Q	Nerve terminals and dendrites, neuroendocrine cells	ω-agatoxin IVA	Neurotransmitter release, dendritic Ca^2+^ transient currents
Ca_v_2.2	N	Nerve terminals and dendrites, neuroendocrine cells	ω-conotoxin CVID, GVIA MVIIA	Neurotransmitter release, Ca^2+^-dependent action potentials
Ca_v_2.3	R	Neuronal cell bodies and dendrites	ω-theraphotoxin-Hg1a(SNX-482)	NeurotransmitterRelease
Ca_v_3.1	T	Neuronal cell bodies and dendrites, cerebellum and thalamus, cardiac and smooth muscles	Pimozide, mibefradil, TTA-P2, Ni^2+^, Zn^2+^	Pacemaking, repetitive firing
Ca_v_3.2	T	CNS: neuronal cell bodiesand dendrites, heart, liver, kidney, lung, skeletal muscle, pancreas	Kurtoxin, pimopzide, mibefradil, Z123212, TTA-P2, Ni^2+^, Zn^2+^	Pacemaking, repetitive firing
Ca_v_3.3	T	CNS: neuronal cell bodies and dendrites	Pimozide, TTA-P2, Zn^2+^ Ni^2+^, mibefradil	Pacemaking, repetitive firing

DHP: Dihydropyridine, PHA: Phenylalkylamine, BTZ: Benzothiazepine, Ni^2+^: Nickel, Zn^2+^: Zinc. Table adapted from: Caterall *et al.*, 2005 [20] and Lewis *et al.*, 2012 [9].

### 2.1. Ca_v_2.2 Channels

The N-type voltage-gated Ca^2+^ channel Ca_v_2.2 is expressed predominantly at presynaptic neuronal terminals throughout the central and peripheral nervous systems, where it is critical for neurotransmitter release. Like the other members of the Ca_v_ family, Ca_v_2.2 is a hetero-oligomeric channel comprising the core pore-forming α1B subunit, which determines the main biophysical and pharmacological properties of the channel, and typically three auxiliary subunits.

### 2.2. Ca_v_2.2 Splice Variants

Alternative splicing is an essential mechanism used extensively in the mammalian nervous system to increase the level of diversity that can be achieved by a set of genes [26]. Two Ca_v_2.2 splice variants have been reported to occur predominantly in the central and peripheral nervous system [27]. The Ca_v_2.2 splice variant 37a is of particular interest, as it replaces the usual variant 37b in a specific subset of rat nociceptive neurons, and may thus represent a potential therapeutic target [28,29,30]. Additional splice variants, named Δ1 and Δ2, lack large parts of the domain II–III linker region including the synaptic protein interaction site, have been isolated from human neuroblastoma cells and brain cDNA libraries [31]. Clinically important, the Δ1 channel was less sensitive to inhibition by both ω-conotoxin MVIIA and GVIA than either the Δ2 variant or the full-length construct [31]. However, since a human splice variant that is only expressed in pathological pain states or in nociceptive pathways has not been identified to date, targeting of Ca_v_ splice variants as an analgesic strategy remains to be validated in humans. 

### 2.3. Ca_v_2.2 Toxin Binding Sites

While it is now appreciated that ω-conotoxins represent some of the most selective known inhibitors of the neuronal Ca_v_2.2 isoform, and many of the key residues involved in binding have been identified, the precise peptide binding determinants and binding sites on Ca_v_ channels are not clearly identified. It is generally accepted that ω-conotoxins act as pore blockers [11,32], although the binding site has been mapped primarily to the external vestibule of the channel in the domain III pore-forming S5–S6 region (see [9] for docking model). In addition, inhibition of Ca_v_2.2 by ω-conotoxins can be notably more complex than would be expected from simple pore blockers sharing a homologous binding site. While some of the best-characterized ω-conotoxins, such as GVIA and MVIIA, bind nearly irreversibly to Ca_v_2.2, reversibility can be induced by voltage protocols which take advantage of the preferential binding of ω-conotoxin to the inactivated rather than resting state of Ca_v_2.2 [33]. 

The large putative extracellular loop between IIIS5 and IIIH5 has been shown to be critically important for the block of Ca_v_2.2 by the extensively studied peptide inhibitor of Ca_v_2.2, ω-conotoxin GVIA (Figure 1). In particular, residues Gln1327, Glu1334, Glu1337, Gln1339 [11] and Gly1326 and Glu1332 of this region were identified as being important for blockage by GVIA [34]. The latter group proposed that Gly1326 may form a barrier that controls access of peptide toxins to the outer vestibule of the channel pore and stabilizes the toxin-channel interaction [34].In addition, the complex between MVIIA or GVIA toxins and Ca_v_2.2 has been proposed to involve a central aromatic residue: tyrosine in the peptide, which is critical for high affinity interactions (Figure 3), plus lateral basic residues that form salt bridges with Glu1332 and perhaps Glu1334 and Glu1337 on the channel [34].

However, voltage-dependent reversibility varies significantly between ω-conotoxins, with CVIE and CVIF showing voltage-dependent reversal particularly in the presence of α2δ1 and β subunits, while reversibility of block by GVIA and MVIIA are only weakly influenced by co-expression with the subunits α2δ1 and β2a or β3 subunit [16,35]. Residues identified to contribute to this reversibility of ω-conotoxin block include in particular Gly1326, as well as intracellular domain II-III linker regions (Figure 1) [31,32]. 

### 2.4. Auxiliary Subunits of Ca_v_ Channels

While the pore-forming α1 subunit determines the main electrophysiological and pharmacological properties of Ca_v_ channels, auxiliary *α2δ* and β-subunits can modify channel gating properties and thus have a significant influence on calcium channel function [35,36]. To date, four auxiliary *α2δ*1–4 subunits, consisting of extracellular disulfide-linked *α2δ* dimer**s** of 170 kDa, and four auxiliary β1–4 subunits [37] forming a 55 kDa cytoplasmic complex with the α1 subunit, have been identified. In addition, a 33 kDa γ subunit comprising four transmembrane segments was first found as a component of skeletal muscle Ca_v_ channels [38], and its related isoforms are expressed in heart and brain (for review see [14,22]). The presence or absence of the auxiliary subunits modulate the α1 subunit function and play an important functional role, modifying and regulating the kinetic as well as pharmacological properties of Ca_v_ channels [16,35,39].

#### 2.4.1. *α2δ* Subunit

The *α2δ* proteins are auxiliary subunits of Ca_v_2.2 that enhance Ca_v_2.2 trafficking and insertion in the plasma membrane [39], but also influence the biophysical and pharmacological properties of the channel (for review see: [40]). A single gene product translates the *α2δ* subunit, which is post-translationally cleaved into the α2 and δ parts that remain associated via disulphide bridges. The α2 protein (~950 amino acids) is entirely extracellular, while the δ part has a small extracellular part that is attached to α2, and a transmembrane domain with a very short cytoplasmic tail [41]. The *α2δ* protein was originally isolated from skeletal muscle as a non-essential subunit of the L-type calcium channel complex [39]. Later it was found to be expressed in many tissues, specifically; the *α2δ* isoforms 1 and 2 are highly expressed by many CNS neurons [42]. Importantly, the isoform 1 is involved in neuropathic pain and is overexpressed after peripheral sensory nerve injury [43,44]. *α2δ*1 and *α2δ*2 are the targets for the gabapentinoid drugs (gabapentin and pregabalin), which are drugs currently used in the treatment of neuropathic pain [44,45,46].

The *α2δ* subunits increase the Ca_v_2.2 inactivation rate to different extents [47]. Specifically, co-expression of *α2δ* subunits has been reported to cause hyperpolarization of the steady-state inactivation as well as an increase in the voltage-dependence [41,47]. Importantly, co-expression of *α2δ* subunit decreases the potency of ω-conotoxins [16,35], which has implications for the therapeutic potential of these peptides. 

Both the physiological functions of *α2δ* subunits and the mechanisms by which binding of gabapentinoid drugs such as gabapentin and pregabalin to *α2δ* subunit translates into therapeutic action are not fully understood. Intriguingly, despite binding to *α2δ* subunits, gabapentin and pregabalin produce little acute inhibition of calcium channel currents. Inhibition of Ca_v_2.2 currents after chronic treatment is generally attributed to down-regulation of Ca_v_2.2 trafficking (for review see [41,47,48]).

Although most of the role of *α2δ*1 in the regulation of pain has been related to regulation of Ca_v_2.2function and trafficking, an alternative analgesic mechanism was proposed recently [49]. The presence of a large extracellular region containing a protein-protein interaction fold, the Von Willebrand Factor A (VWF-A) domain, suggests that the *α2δ*1 subunit could serve as a receptor for extracellular ligands itself [41,49]. Indeed, it has been reported that the VWF-A domain of the *α2δ*1 subunit binds to proteins of the thrombospondin family [49]. Thus, *α2δ*1 is proposed to be the neuronal thrombospondin receptor which is involved in CNS synaptogenesis (synapse formation) and synaptic maturation [49]. The *α2δ*1 inhibitor gabapentin was also found to disrupt the interaction of the *α2δ*1 subunit with proteins of the thrombospondin family, thus leading to inhibition of synapse formation [49]. This occurred both *in vitro* and *in vivo* when neonatal mice were treated with gabapentin [47,49], and it has been proposed that inhibited synapse formation represent an additional mechanism by which *α2δ*1 inhibitors produce analgesia [49].

#### 2.4.2. β Subunit

Four different genes encode the β subunits (β1–β4) and numerous splice variants are known [39]. The β subunit is the only subunit of the channel that is entirely cytosolic. It has been proposed that these subunits associate with the α1 subunit predominantly through a highly conserved high affinity interaction that is mediated by the Alpha Interaction Domain (AID) in the α1 subunit [50] and a corresponding Beta Interaction Domain (BID) in the β subunit [51]. The β subunit aids the trafficking of α1 to the plasma membrane. This was initially thought to occur due to its ability to mask an endoplasmic reticulum retention signal in the α1 subunit domain I-II linker [51,52]. However, several recent studies have reported data which is inconsistent with this hypothesis (for review see [53]). Transplanting the I–II linker of the Ca_v_2.2 α1 subunit into Ca_v_3.1, which does not require the β subunit for its function, caused current up-regulation rather than down-regulation [54]. Similarly, domain I–II linkers from several different Ca_v_α1 subunits do not cause ER retention of CD8 or CD4 reporter protein [55,56]. In addition, several studies have implicated regions other than the I–II linker in Ca_v_α1 trafficking [56,57,58], and another study suggested that the mechanism of β subunit-mediated Ca_v_ trafficking involves proteosomal degradation [59].

β subunit co-expression has a large effect on the level of expression, voltage dependence and kinetics of gating of cardiac and neuronal Ca_v_ channels. In general, the level of Ca_v_expression is increased, the voltage dependence of activation and inactivation is shifted to more negative membrane potentials, and the rate of inactivation is increased. However, these effects are β subunit specific [22], with the β2 subunit slowing channel inactivation in combination with α1B-b/*α2δ*1, while the β3 induces faster inactivation in combination with α1B-b/*α2δ*1 [16]. Recovery from ω-conotoxins block is also influenced to varying degrees by the different β subunit isoforms co-expressed with α1B-b/*α2δ* subunits [8,16]. 

#### 2.4.3. γ Subunit

The γ subunit was originally known to only be associated with the skeletal muscle voltage-gated channel complex. However, recently, expression of γ isoforms 2 and 3 were established in the brain [60,61] and genetic studies revealed the existence of a γ subunit isoform in the brain whose lack of expression is responsible for the epileptic and ataxic phenotype of the stargazer mouse [61]. In addition, the γ subunit has been found as part of a neuronal membrane complex with Ca_v_1.2 [62]. The γ subunits share a conserved four transmembrane domain topology, with predicted intracellular amino and carboxy termini, and a consensus site for cAMP/cGMP phosphorylation [39]. Although the effects of auxiliary γ subunits on the pharmacology of Ca_v _channels have not been extensively studied, a γ isoform-dependent negative effect on Ca_v_3.1 low voltage-activated current density has been described [63]. In addition, patch-clamp recordings showed that transient transfection of γ1 drastically inhibited macroscopic currents through recombinant N-type calcium channels (Ca_V_2.2/*α2δ*–1/β3) expressed in HEK-293 cells [64].

### 2.5. Ca_v_2.2 Channels as Analgesic Target

**Figure 2:**
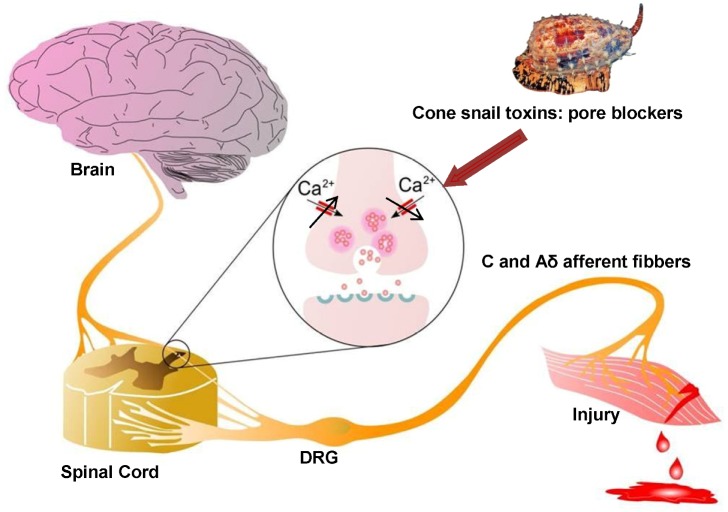
**Role of Ca_v_2.2 in ascending pain pathway**: Pain signals originating from peripheral C and Aδ afferent fibers evoke Ca_v_2.2-mediated synaptic vesicle release of neurotransmitters such as glutamate, substance P, and CGRP which activate spinal neurons, altering sensory excitability and leading to pain sensations. Direct block of Ca_v_2.2 channels by ω-conotoxins from cone snail venoms stops the link between the origin of pain and the transmission of pain sensation to the brain, because it decreases excessive calcium signalling during hyperactive excitation. Figure adapted from Zamponi *et al.* [67].

Ca_v_2.2 is expressed in a common pathway downstream from the large variety of receptors that mediate pain responses, thus, inhibition of this Ca_v_ subtype can mediate analgesia [65,66]. Although different types of calcium channels are found in nociceptive pathways, Ca_v_2.2 is particularly important in controlling signaling in nociceptive pathways such as the ventral and dorsal horn of the spinal cord and dorsal root ganglion (DRG) neurons, especially along the dendrites and at presynaptic terminals where it contributes critically to neurotransmitter release [67]. As a consequence of the change in membrane potential that occurs in response to a painful peripheral stimulus, Ca_v_2.2 channels open, resulting in an increase in intracellular calcium. This in turn triggers synaptic vesicle release of neurotransmitters such as glutamate, substance P and CGRP (Calcitonin Gene Related Peptide), which activate post-synaptic receptors in the membrane of spinothalamic neurons and nerve terminals localized in the dorsal horn of the spinal cord [67,68] (Figure 2), allowing the propagation of pain signals.

Several lines of evidence support Ca_v_2.2 as an important pain target. Studies of Ca_v_2.2 knock-out mice have shown that these animals, in contrast to Ca_v_2.1 knock-out mice, had normal CNS (central nerve system) and motor function, but were resistant to development of neuropathic pain in a spinal nerve ligation model**,** and were insensitive to formalin-induced or visceral pain [69,70]. Furthermore, morphine, an opioid analgesic used for many years as the first option to treat severe pain, indirectly modulates Ca_v_2.2 channels. Binding of morphine to μ-opioid receptors leads to inhibition of Ca_v_2.2 through Gβγ-mediated signaling that reduces the ability of DRG sensory neurons to propagate pain signals centrally [67,71]. 

In addition, the *α2δ*1 auxiliary subunit of the Ca_v_2.2 channels has been reported to be up-regulated in pain states in the dorsal root ganglion (DRG) and spinal dorsal horn after nerve injury [43,44,72,73], suggesting an involvement of this subunit with pathophysiological mechanisms of pain [73]. Although as discussed above, other mechanisms may underlie the involvement of the *α2δ* subunit with pain; studies using transgenic mice have found that the pro-algesic effects of *α2δ* subunits are mediated at least partially by enhancing Ca_v_2.2 activity in sensory neurons [73]. In this study the author suggested it occurred possibly through enhanced formation of the functional Ca_v_ complex in lipid raft micro domains, as well as through hyper-excitability in dorsal horn neurons in response to peripheral stimulation [73].

Lastly, in 2004 the Ca_v_2.2 blocker peptide ω-conotoxin MVIIA or ziconotide (Prialt^®^), was approved for the treatment of severe chronic pain associated with cancer, AIDS and neuropathies. Intrathecal injection (delivered directly to the spinal cord) of this synthetic peptide has proved effective against both neuropathic and inflammatory pain in laboratory animals and man [15,18,69,74,75], although associated side effects limit its application. Importantly, ziconotide acts synergistically with opioid analgesics without inducing tolerance or addiction [19].

### 2.6. Venoms as a Rich Source of Ca_v_2.2 Channel Blockers

Cone snails comprise over 500 species of marine predatory gastropods that are mostly found on or near coral reefs in tropical and subtropical waters, including the coastal waters of Australia. They produce a highly complex mixture of venom peptides which have evolved for prey capture and defense (for review see [1,9]). This includes the ω-conotoxins, small disulfide-rich peptides with defined activity at mammalian Ca_v_ isoforms. 

The ω-conotoxins belong to the O-superfamily, which also includes the δ-conotoxins (inhibit the fast inactivation of the voltage gated Na^+ ^channels), µO-conotoxins (inhibit voltage-gated Na^+ ^currents) and κ-conotoxins (interact with K^+^ channels). To date, ω-conotoxins targeting mammalian Ca_v _isoforms have only been isolated from piscivorous cone snails, where they are likely to have evolved as part of the “motor cabal” leading to rapid flaccid paralysis of their fish prey [76]. In contrast, the few ω-conotoxins isolated from mollusc-hunting species to date (PnVIA and PnVIB) have been found to be inactive at mammalian Ca_v _channels, suggesting distinct phylum-selective pharmacology, consistent with their sequence diversity (for review see: [9]).

**Figure 3:**
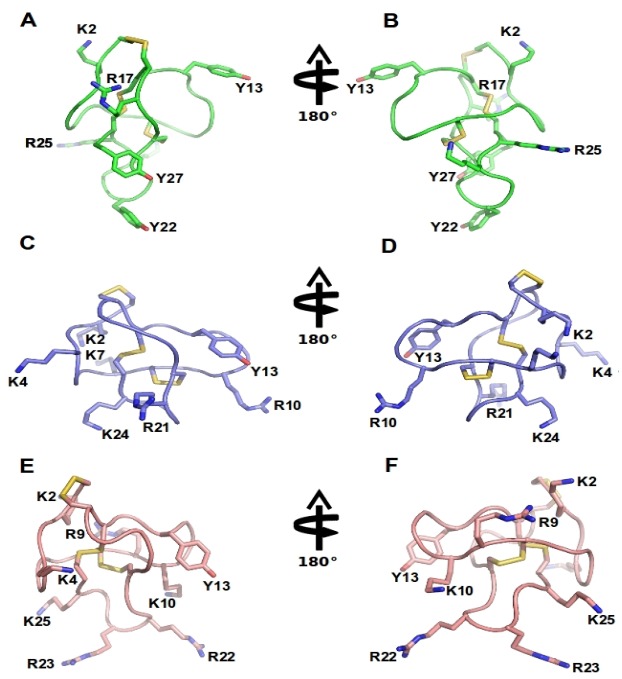
ω-Conotoxins structure: NMR structure of GVIA (PDB 1TTL, green A-B), MVIIA (PDB 1 TTK, blue C-D) and MVIIC (PDB 1CNN, pink D-E). Represented are two different orientations. Disulfide bridges are shown in yellow and important amino acid residues, including Y13 (tyrosine13) and K2 (lysine2) and several positively charged residues exposed in the side chain are labeled.

The ω-conotoxin family comprises peptides ranging from 24 to 30 amino acids in length. As seen for the snake and spider venom toxins, the relatively low sequence homology among ω-conotoxins suggests that the overall three-dimensional structure and charge distribution underpin their interaction with Ca_v_2.2 channels (Table 2, Figure 3). Although the remainder of the amino acids shows no absolute sequence conservation, positions **2 **and **25 **are always occupied by either a lysine (K) or an arginine (R), and the most active forms have a tyrosine (Y) at position **13**. Moreover, a high proportion of residues contain hydroxyl moieties, which is accentuated in many of the ω-conotoxins by the substitution of γ-hydroxyproline for proline [77]. Disulphide bonds fold the peptide into a highly conserved cysteine framework pattern (C-C-CC-C-C) (Table 2, Figure 3) that contributes to tertiary structure stabilization. This configuration defines the canonical ω-conotoxin fold, which comprises a triple-stranded β-sheet/inhibitory cysteine-knot framework (Figure 3) [78]. A unique feature of conotoxins is their high degree of post-translational modification [79], and several ω-conotoxin have stability enhanced naturally through the use of these post-translational modifications (PTMs) [1,80]. This mechanism may limit potential degradation by carboxylases, which would otherwise rend the peptide biologically inactive. In addition, a C-terminal amide and the relative abundance of basic residues within ω-conotoxins class gives them an overall net positive charge, which presumably assists in their complementary binding to Ca_v_2.2 channels [81].

**Figure 4:**
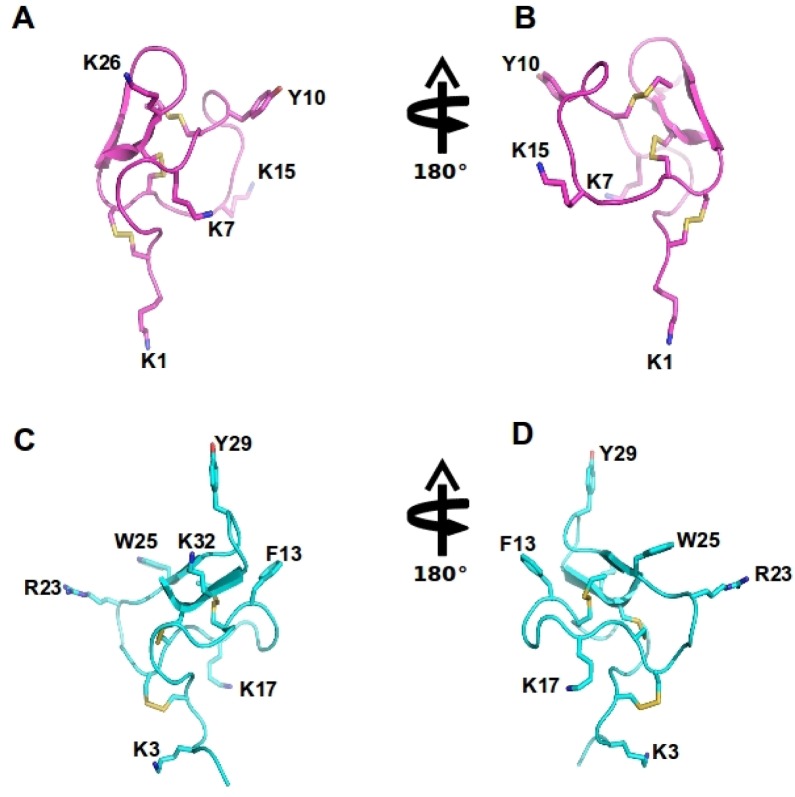
Spider ω-theraphotoxin-Hh1a (HWTX-X) and Ptu1 structure: The structure of huwentoxin 10 (HWTX-X) (pink A–B) and Ptu1 (blue C–D) showing two different orientations. The position of the four loops is indicated, and disulfide bridges are shown in yellow. Important amino acid residues described to have similar function to tyrosine13 and lysine 2 in the ω-conotoxins are represented, including Y10 and K7 in Ptu1, as well as F13 (phenylalanine13) and K17 (lysine17) and several positively charged residues exposed in the side chain are labeled.

The binding determinants for the high affinity interaction of ω-conotoxins with Ca_v_2.2 have been proposed to rely on a two-point pharmacophore formed by the highly conserved Y13 (tyrosine) and K2 (lysine) [8,85,86,93,94]. However, Y13 and K2 are also often conserved in ω-conotoxins with activity at Ca_v_2.1. Thus, residues contributing to selectivity at Ca_v_2.2 over Ca_v_2.1 are less clear. Intriguingly, the two ω-conotoxins that display most sequence homology, MVIIA and MVIIC, target quite different Ca_v_ subtypes (Ca_v_2.2 and Ca_v_2.1, respectively), whereas conversely, ω-conotoxins GVIA and MVIIA inhibit the same Ca_v_ subtype (Ca_v_2.2), despite significantly lower sequence homology (Table 2, Figure 4). An extensive study assessing loop splice hybrids found that selectivity of MVIIA and MVIIC for Ca_v_2.2 and Ca_v_2.1 respectively, is controlled in a concerted manner by residues of loop 2 and 4 (Figure 3) [81]. Peptides with homogeneous combinations of loop 2 and 4 display clear selectivity while those with heterogeneous combinations of loops 2 and 4, are less discriminatory (Figure 3A−B) [81]. CVID is notable for its high potency at Ca_v_2.2 and low potency at Ca_v_2.1, making this peptide the most Ca_v_2.2-selective peptide described to date [8,90].

**Table 2:** ω-ConotoxinCa_v_2.2 blockers: Sequence, indicating conserved cysteine residues in bold face type and potency at ^125^I-GVIA or MVIIA binding assays.

ω-conotoxin name	ω-conotoxin Sequence	^125^I-Ctx binding assays to rat brain IC_50_/K_d _(nM)	Reference
CnVIIA	**C**KGKGAO**C**TRLMYD**CC**HGS**C**SSSKGRC*	0.4 (2.2 > 2.1)	[82]
CVIA	**C**KSTGAS**C**RRTSYD**CC**TGS**C**RSGR**C**	0.6 (2.2 > 1.2)	[8]
CVIB	**C**KGKGAS**C**RKTMYD**CC**RGS**C**RSGR**C**	7.7 (2.2~2.1 > 2.3)	[8]
CVIC	**C**KGKGQS**C**SKLMYD**CC**TGS**C**-SRRGK**C**	7.6 (2.1~2.2)	[8]
CVID	**C**KSKGAK**C**SKLMYD**CC**SGS**C**SGTVGR**C**	0.07 (2.2 > 2.1)	[8]
CVIE	**C**KGKGAS**C**RRTSYD**CC**TGS**C**RSGR**C**	0.025 (2.2 > 2.1 > 1.2~1.3~2.3	[16]
CVIF	**C**KGKGAS**C**RRTSYD**CC**TGS**C**RLGR**C**	0.098 (2.2 > 2.1 > 1.2~1.3~2.3)	[16]
FVIA	**C**KGTGKS**C**SRIAYN**CC**TGS**C**RSGK**C**	ND (2.2 > 2.1 > 3.2)	[83]
GVIA	**C**KSOGSS**C**SOTSYN**CC**RS**C**NOYTKR**C**Y*	0.04 (2.2 > 2.1)	[84,85,86,87,88]
GVIB	**C**KSOGSS**C**SOTSYN**CC**R-S**C**NOYTKR**C**YG*	ND	[88,89]
GVIIA	**C**KSOGTO**C**SRGMRD**CC**TS**C**LLYSNK**C**RRY*	3.7 (ND)	[88,89,90]
GVIIB	**C**KSOGTO**C**SRGMRD**CC**TS**C**LSYSNK**C**RRY*	ND	[88]
MVIIA	**C**KGKGAK**C**SRLMYD**CC**TGS**C**RSGK**C**	0.055 (2.2 > 2.1)	[13,81,87]
RVIA	**C**KPPGSP**C**RVSSYN**CC**SS**C**KSYNKK**C**G	0.25 (2.2)	[10]
TVIA	**C**LSXGSS**C**SXTSYN**CC**RS**C**NXYSRK**C**R	ND (2.2 > 2.1)	[91,92]

Source: Conoserver database: www.conoserver.org. ^125^I-Ctx = ^125^I-GVIA or ^125^I-MVIIA displacement assays to define ω- conotoxins binding to Ca_v_2.2 expressed in different brain preparations including rat, chicken and mouse brain. ND= Not determined; in brackets the order of Ca_v_ type selectivity for each ω-conotoxin is described.* O=hydoxyproline (PTM: post-translational modification).

Importantly, it has been reported that the affinity of ω-conotoxins is often profoundly affected by the presence of auxiliary subunits, in particular α2δ and β subunits [16,35,95,96,97,98]. However, the degree to which co-expression of auxiliary subunits affects ω-conotoxin potency can vary significantly, with MVIIA, GVIA, CVID and CVIF being particularly susceptible to affinity reductions in the presence of α2δ1 subunits, while the potency of CVIE is affected to a lesser degree by co-expression with auxiliary subunits [16,35]. Such pharmacological effects can have profound implications for the therapeutic potential of ω-conotoxins, as α2δ1 subunit expression is increased in dorsal root ganglion and spinal cord in several animal models of neuropathic pain [1,16,99].

#### 2.6.1. Cone Snail Venom Peptide Ca_v_2.2 Inhibitors for the Treatment of Pain

While both direct and indirect inhibition of Ca_v_2.2 by toxins and small molecules is a clinically validated analgesic strategy, currently available peptide Ca_v_2.2 inhibitors suffer from limitations that restrict their more widespread use. These limitations include the need for intrathecal administration for effective delivery to spinal sites of action. In addition, dose-limiting side effects including dizziness, nystagmus, somnolence, abnormal gait and ataxia lead to a narrow therapeutic index. Thus, since its approval in 2004, ziconotide remains the only peptidic Ca_v_2.2 inhibitor approved for the treatment of severe refractory pain. The precise mechanisms underlying this unfavorable side effect profile is not entirely clear. Contributing factors most likely include a lack of *in vivo* selectivity over other Ca_v_ subtypes (although the *in vitro* binding selectivity is exceptional), inhibition of Ca_v_2.2 at supraspinal sites [15], inhibition at inhibitory interneurons or descending inhibitory synapses [57], or pharmacodynamic effects such as a slow off-rate and poor affinity for Ca_v_2.2 co-expressed with auxiliary subunits [100].

#### 2.6.2. Effect of Selectivity on Side Effect Profile

While Ca_v_2.2 is important in mediating synaptic transmission at nociceptive synapses, contributions from other Ca_v_ channel subtypes, most notably Ca_v_2.1 and Ca_v_2.3, also mediate significant neurotransmitter release at neuronal synapses [101,102]. Thus, non-selective block of these Ca_v_ isoforms can contribute to severe side effects arising from inhibition of neurotransmitter release in non-nociceptive neurons. Accordingly, a significant challenge in targeting Ca_v_2.2 for therapeutic drug discovery is likely to be selectivity over other Ca_v_ subtypes, especially Ca_v_2.1, which is highly homologous to Ca_v_2.2. However, the residues lining the pore in all S5 and S6 segments, which are proposed to contain the binding sites for most of the therapeutically useful drugs that block voltage-gated calcium channels, are nearly identical [103]. Thus, many venom peptides with activity at Ca_v_2.2 also inhibit Ca_v_2.1 to varying degrees, and *vice versa*. Systemically administered small molecule or peptide inhibitors of Ca_v_2.2, while efficacious, lead to additional side effects resulting predominantly from action on the cardiovascular system [104]. However, recent data from animal models provide some evidence that a better therapeutic margin may be achievable with the ω-conotoxins. For example, intravenously administered leconotide (CVID, AM336), the most selective N-type blocker described to date [8], has shown efficacy in animal models of bone cancer pain [8]. Accordingly, additional ω-conotoxins are currently undergoing pre-clinical and clinical trials, and novel ω-contoxins with improved safety margin will hopefully reach the clinic in the future. 

### 2.7. Ca_v_2.2 Inhibitor Toxins from Spiders

Spiders, the most species-rich family of terrestrial venomous predators, have evolved highly complex venoms to assist with prey capture ([105], for review see [106]). Like cone snails, spiders have evolved a myriad of peptide venom components with activity at voltage-gated ion channels including Ca_v_2.2 (for review see [106,107]). 

Interestingly, spider toxins have provided some of the most subtype-selective Ca_v_2.1 and Ca_v_2.3 inhibitors known to date [108,109]. However, in contrast to conotoxins, which are notable for their selectivity for Ca_v_2.2 in particular (for review see [9]), relatively few spider venom peptides are active at Ca_v_2.2, and even fewer show selectivity for Ca_v_2.2 over other Ca_v_ isoforms (see Table 2 and Table 3) [4,5].

**Table 3:** **Ca_v_2**.2 inhibitors from spider toxins.

Toxin name/Synonym	Functional (IC_50_)/Binding (K_d_) at Ca_v_2.2	Amino acid sequence	Reference
μ/ω-theraphotoxin-Hh1a/Huwentoxin-1	100 nM (ND)	ACKGVFGACTPGKNECCPNRVCSDKHKWCKWKL	[110,111]
μ/ω-theraphotoxin-Hh1b/Huwentoxin1a3	(ND)	ACKGVFGACTPGKNECCPNRVCSDKHKWCKWKL	[112]
μ/ω-theraphotoxin Hh1c/Huwentoxin1a10	(ND)	ACKGVFDACTPGKNECCSNRVCSDKHKWCKWKL	[112,113]
μ/ω-theraphotoxin-Hh1d/Huwentoxin-1a6	(ND)	ACKGVFDACTPGKNECCPNRVCSDEHKWCKWKL	[112]
ω-agatoxin-Aa2a/ω-agatoxin IIA	10 nM (Y)	GCIEIGGDCDGYQEKSYCQCCRNNGFCS	[114,115]
ω-agatoxin-Aa3a/ω-agatoxin IIIA	1.4 nM/170 pM (N)	SCIDIGGDCDGEKDDCQCCRRNGYCSCYSLFGYLKSGCKCVVGTSAEFQGICRRKARQCYNSDPDKCESHNKPKRR	[116,133]
ω-agatoxin-Aa3b/ω-agatoxin IIIB	140 nM/2.4 nM (N)	SCIDFGGDCDGEKDDCQCCRSNGYCSCYNLFGYLKSGCKCEVGTSAEFRRICRRKAKQCYNSDPDKCVSVYKPKRR	[116,117]
ω-agatoxin-Aa3d/ω-agatoxin IIID	35 nM (N)	SCIKIGEDCDGDKDDCQCCRTNGYCSXYXLFGYLKSG	[116]
ω-agatoxin-Aa3f/ω-agatoxin IIIA (58T)	1.4 nM (N)	SCIDIGGDCDGEKDDCQCCRRNGYCSCYSLFGYLKSGCKCVVGTSAEFQGICRRKARTCYNSDPDKCESHNKPKRR	[116]
ω-agatoxin-Aa3g/ω-agatoxin IIIB (35R)	2.4 nM (N)	SCIDFGGDCDGEKDDCQCCRSNGYCSCYNLFGYLRSGCKCEVGTSAEFRRICRRKAKQCYNSDPDKCVSVYKPKRR	[116]
ω-agatoxin-Aa3h/ω-agatoxin IIIB (29S)	2.4 nM (N)	SCIDFGGDCDGEKDDCQCCRSNGYCSCYSLFGYLKSGCKCEVGTSAEFRRICRRKAKQCYNSDPDKCVSVYKPKRR	[116]
ω-ctenitoxin-Pn2a/Neurotoxin Tx3–3	>320nM/50 pM (N)	GCANAYKSCNGPHTCCWGYNGYKKACICSGXNWK	[7,118,119]
ω-ctenitoxin-Pn3a/Neurotoxin Tx3–4	50 pM (N)	SCINVGDFCDGKKDDCQCCRDNAFCSCSVIFGYKTNCRCEVGTTATSYGICMAKHKCGRQTTCTKPCLSKRCKKNH	[119]
ω-ctenitoxin-Pn4a/Neurotoxin Tx3–6 PnTx3–6/Phα1β	122 nM (N)	ACIPRGEICTDDCECCGCDNQCYCPPGSSLGIFKCSCAHANKYFCNRKKEKCKKA	[119,120]
ω-ctenitoxin-Pr1a/Neurotoxin PRTx3–7	>1000 nM (N)	ACAGLYKKCGKGVNTCCENRPCKCDLAMGNCICKKKFVEFFGG	[121,122]
ω-segestritoxin-Sf1a/SNX-325	~10 nM (Y)	GSCIESGKSCTHSRSMKNGLCCPKSRCNCRQIQHRHDYLGKRKYSCRCS	[123]
ω-theraphotoxin-Hh1a/Huwentoxin-10	40 nM (Y)	KCLPPGKPCYGATQKIPCCGVCSHNKCT	[113]

Source: Arachnoserver spider venom database: http://www.arachnoserver.org [4,5]. Selective for Ca_v_2.2 channel? (Y) = yes, (N) = no, ND = Not determined; Binding: ^125^I-Ctx performed in different brain preparations, including rat, chicken and mouse brain.

**Figure 5:**
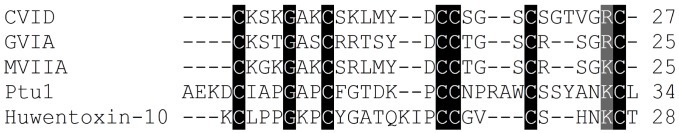
Amino acid sequence alignment of the Ca_v_2.2 inhibitor toxins, ω-conotoxins from cone snails, spiders and the assassin bug *Peirates turpis*. Cysteines common to all toxins which are important for these peptides extraordinary stability are highlighted, in addition to positively charged amino acids suggested to be important for binding of these toxins to Ca_v_2.2 channels.

Spider venom peptides with activity at Ca_v_2.2 have to date only been described from *Haplopelma huwenum, Agelenopsis aperta, Phoneutria nigriventer, Phoneutria reidyi* and *Segestria florentina* [7,110,111,112,113,114,115,116,117,118,119,120,121,122,123]. These Ca_v_2.2 inhibitors are structurally diverse and can share common structural motifs with the ω-conotoxins (Figure 3, Figure 4, Figure 5), with 3 disulfide bonds forming an inhibitory cysteine knot, or have as many as 7 disulfide bonds, as is the case for ω-ctenitoxin-Pn3a [113,119]. The majority of these peptides have little selectivity for Ca_v_2.2 and displays activity at Ca_v_1, Ca_v_2.1 and Ca_v_2.3 isoforms. For example, ω-agatoxin-Aa3a is equipotent at Ca_v_1 and Ca_v_2.2, and ω-ctenitoxin-Pn2a from *Phoneutria nigriventer* blocks voltage-gated calcium channels Ca_v_2.1, Ca_v_2.3, Ca_v_1 and Ca_v_2.2 in decreasing order of potency [119]. Some spider venom peptides, such as the theraphotoxins Hh1a-d, even inhibit the related Na_v_ channels and are designated as ω-/µ-toxins based on this pharmacological profile [110]. In contrast, ω-segestritoxin-Sf1a, ω-agatoxin-Aa2a and ω-theraphotoxin-Hh1a or huwentoxin-10 preferably inhibit vertebrate Ca_v_2.2, although it is unclear whether their selectivity can match the more than 10,000-fold preference for Ca_v_2.2 over Ca_v_2.1 exhibited by some conotoxins [8,113,115,123]. Given the similar overall structure shared by cone snail and spider venom peptides, these divergent selectivity profiles are surprising. However, as illustrated by conopeptides, small differences in structure may result in profound effects on Ca_v_ selectivity. Specifically, conotoxins MVIIA, MVIIC, GVIA and CVID share a high degree of sequence similarity and are structurally closely related. Nonetheless, MVIIC is a selective Ca_v_2.1 inhibitor, while MVIIA, GIVA and CVID are highly selective Ca_v_2.2 blockers [8].

Though spider toxins are generally accepted to act as gating modifiers at Ca_v_ channels, relatively little is known about the mechanism of action of Ca_v_2.2 block. For example, ω-ctenitoxin-Pn4a was shown to decrease peak Ca_v_2.2 current with little effect on voltage-dependence of activation [120], and Ca_v_2.2-specific inhibitors from spider venoms such as ω-segestritoxin-Sf1a were able to displace radiolabelled ω-conotoxins (Table 3) [123], suggesting that these peptides may act like pore blockers rather than gating modifiers at Ca_v_2.2. Importantly, little is known about the influence of Ca_v_ auxiliary subunits on inhibition by spider venom peptides. Given that the pharmacology of cone snail toxins is known to be affected by auxiliary subunits [16,35,124], future studies should include characterising the effect of auxiliary subunits on inhibition of Ca_v_2.2 by spider venom peptides.

#### 2.7.1. ω-Agatoxin-Aa2a

The venom of *Agelenopsis aperta* provided the first source of Ca_v _inhibitors, making the agatoxins some of the best-studied spider Ca_v _channel antagonists. Based on their structural homology and pharmacological properties, agatoxins have been classified into four distinct groups (agatoxins I –IV). While type I and III agatoxins are selective for Ca_v_1 and Ca_v_2.1, respectively, type II and III agatoxins display activity at Ca_v_2.2. However, while type III agatoxins such as ω-agatoxin-Aa3a are active at all high-threshold Ca_v_ channel isoforms, including Ca_v_2.1, Ca_v_2.2, Ca_v_2.3 and Ca_v_1, type II agatoxins target Ca_v_2.2 over other Ca_v_ isoforms [114,115]. ω-Agatoxin-Aa2a, an 11 kDa mature toxin comprised of 92 residues, displaced ω-conotoxin GVIA binding and synergistically blocked neurotransmitter release with the unrelated L-type toxin ω-AGTX-Aa1a [114]. While more detailed selectivity studies have not been carried out, this suggests that the toxin targets primarily Ca_v_2.2 channels [115].

The structural requirements for high affinity inhibition of Ca_v_2.1 by type IV agatoxins such as ω-agatoxin-Aa4a have been relatively well defined, and are proposed to involve a positively charged area, formed by several basic amino acid residues near the hydrophobic C-terminus [125], as well as a crucial tryptophan residue in position 14. In contrast, nothing is known about the structure-activity of ω-agatoxin-Aa2a. Thus, future studies are necessary to improve our understanding of the molecular interaction between ω-agatoxin-Aa2a and Ca_v_2.2.

#### 2.7.2. ω-Theraphotoxin-Hh1a

ω-Theraphotoxin-Hh1a (huwentoxin 10 or HWTX-X) was isolated from the venom of the Chinese bird spider and shares several properties with the ω-conotoxins [113]. In contrast to ω-conotoxins, the C-terminus of HWTX-X is not amidated (Table 3, Figure 5) [113].This relatively small 28 residue peptide is stabilized by three disulfide bonds, and shares a functional motif, defined by a critical aromatic residue and several basic residues, with the ω-conotoxin GVIA (Figure 4). Intriguingly, huwentoxin 10 was unable to inhibit twitch responses of electrically stimulated rat vas deferens, suggesting selectivity for different Ca_v_2.2 or splice variants [113]. While the analgesic potential of ω-theraphotoxin-Hh1a has not been assessed to date, intraperitoneal injection in mice produced no toxic effects [113], suggesting that this peptide could be a promising therapeutic lead for the treatment of pain.

#### 2.7.3. ω-Segestritoxin-Sf1a

ω-Segestritoxin-Sf1a (SNX-325) has been described as a selective Ca_v_2.2 inhibitor, based on its ability to inhibit Ca_v_2.2 responses in oocytes as well as KCl-mediated neurotransmitter release in hippocampal slices [123]. Interestingly, although ω-segestritoxin-Sf1a shares little structural homology with the ω-conotoxins and is a large peptide stabilized by 4 disulfide bonds, it shares a common binding site with the ω-conotoxins and was able to displace radiolabelled MVIIA from a rat brain synaptosome preparation [123].

### 2.8. Spider Venom Ca_v_2.2 Inhibitors in Pain

While the analgesic potential of Ca_v_2.2-selective peptides from spider venom has not been assessed extensively, several studies report efficacy with little side effects in various animal models of pain. ω-ctenitoxin-Pn4a (PnTx3–6, Phα1β, neurotoxin 3–6), a toxin from the spider *Phoneutria nigriventer* which non-selectively inhibits neuronal Ca_v _with a rank order of potency of Ca_v_1.2 > 2.2 > 2.1 > 2.3 [119,120,126,127], elicited prolonged analgesia after intrathecal administration in an animal model of incisional pain [128,129]. ω-ctenitoxin-Pn4a and had no effect on mean arterial blood pressure, heart rate or gross neuronal performance, suggesting that Ca_v_ inhibitors from spider venoms could also find therapeutic application for the treatment of pain [128,129]. Another non-selective toxin from *Phoneutria* *nigriventer,* ω-ctenitoxin-Pn2a (rank order of potency: Ca_v_2.1 > 2.3 > 1 > 2.2) [118], showed prevalent antinociceptive effects in neuropathic pain models and did not cause adverse motor effects efficacious doses [130]. However, as Ca_v_2.1 and Ca_v_3 have been proposed as analgesic targets in their own right [131,132], the contribution of non-Ca_v_2.2 channels to these observed *in vivo* effects remains to be determined. 

## 3. Ca_v_2.2 Inhibitors from Other Venomous Animals

Ca_v_ modulators are also found in the venom of other venomous species, including snakes, scorpions, and centipedes. However, these peptides, including calcicludine from the green mamba [134] and kurtoxin from the venom of the scorpion *Parabuthus transvaalicus* [135], show no selectivity for Ca_v_2.2, or in the case of glycerotoxin [136] from the marine blood worm *Glycera convulata*, are Ca_v_2.2 enhancers and are thus included here only for completeness. 

### 3.1. Scorpion Venom Peptides

Kurtoxin, a 63-residue peptide isolated from the venom of the scorpion *Parabuthus transvaalicus* is related to the α-scorpion toxins, a family of toxins that slow inactivation of Na_v _channels. While kurtoxin has shown selectivity for heterologous expressed Ca_v_3 or T-type calcium channels [137], activity at Ca_v_2.2 has been described in rat sympathetic and thalamic neurons [135]. In contrast to ω-conotoxins with activity at Ca_v_2.2 channels, scorpion toxins with activity at Ca_v_ channels, including kurtoxin, act as gating modifiers. Thus, selectivity differences observed in overexpression systems compared to neurons may be due to the influence of auxiliary subunits, although this has not been assessed to date.

### 3.2. Snake Venom Peptides

Calcicludine (60 residues, 3 disulfide bonds) and calciseptine (60 residues, 4 disulfide bonds) were isolated from the venom of the black mamba, *Dendroaspis polyepsis*, and the green mamba, *Dendroaspis angusticeps*, respectively. While calciseptine inhibits L-type calcium channels and has no activity at Ca_v_2.2 [138], calcicludine is less selective and potently inhibits all high-voltage-activated calcium channels, including Ca_v_2.2 [139].

### 3.3. Centipede Venom Peptides

Recently, two peptides with activity at neuronal Ca_v _channels were isolated from the venom of the centipede *Scolopendra subspinipes mutilans*. ω-SLPTX-Ssm1a, an 83 residues peptide containing 7 cysteines was found to act as an activator of Ca_v_ channels [140], while a smaller peptide, ω-SLPTX-Ssm2a (54 residues) was shown to inhibit Ca_v_ channels expressed in DRG neurons [140]. However, the Ca_v_ subtype selectivity of these peptides is currently unknown but it may include Ca_v_2.2, which is expressed in peripheral sensory neurons.

### 3.4. Assassin Bug Toxins

The predatory assassin bugs (Hemiptera: Reduviidae) contain a complex mixture of small and large peptides in its toxic saliva which is used to immobilize and pre-digest their prey, and for defense against predators [141]. Three novel peptide toxins, named Ado1, Ptu1 and Iob1, isolated from three species of assassin bugs (*P. turpis*, *A. dohrni*, and *I. obscurus*), were biologically active in electrophysiological assays using BHK-N101 cells stably expressing rabbit Ca_v_2.2, β_1A_ subunit, and *α2δ* subunit [141]. Ptu1 binds reversibly to Ca_v_2.2 with lower affinity than ω-conotoxin MVIIA. Ptu1 lacks most of the residues shown to be important for ω-conotoxin binding to the N-type calcium channel, including equivalents of Tyr13 or Lys2 (Figure 4 and Figure 5). This peptide belongs to the inhibitory cysteine knot structural family (ICK) that consists of a four-loop Cys scaffold forming a compact disulfide-bonded core [142]. Thus, as for the other venom peptides compared here, the structure of Ptu1 aligned with related Ca_v_2.2 blockers MVIIA and GVIA, indicating that a common functional motif can be supported by a common three-dimensional structure despite the lack of sequence homology (Figure 5). 

## 4. High Throughput Assays for Novel Ca_v_2.2 Channel Inhibitors

Venoms represent complex natural compound libraries which have evolved over millions of years, and contain some of the most subtype-selective ion channel modulators known. Assay-guided fractionation, the process whereby bioactive components from venom are isolated based on sequential rounds of fractionation and activity testing, has been used as a successful strategy for the discovery and isolation of novel venom components for many years (for review see [143]). This process requires sensitive, accurate and robust assays which are able to detect activity at the biological target of interest.

Electrophysiological techniques are generally considered the “gold standard” technique to study ion channels, including Ca_v_2.2 channels. However, classical electrophysiological recordings are labour-intensive and generally low-throughput, while electrophysiology in high throughput format for primary drug screening is difficult and/or costly to implement [98,144]. Therefore, incorporation of functional cell-based high-throughput screening (HTS) assays can significantly speed up the identification of novel candidates from large compound libraries [144,145], such as animal venoms.

A range of HTS assays to screen new Ca_v_2.2 channel inhibitors have been described, including fluorescence-based Ca^2+^ assays and radioligand binding assays [124,144,145,146,147]. Radioligand binding assays are amenable to HTS, however, these assays cannot detect modulators acting at different sites from the ω-conotoxin site on Ca_v_2.2. Given the narrow safety window of pore blocking peptides such as ziconotide, small molecule inhibitors of Ca_v_2.2 channels with state-dependent blocking activity may provide improved therapeutic margins (for review see: [148]). In addition, the membrane potential as well as association of the pore-forming α subunit of Ca_v_2.2 with auxiliary subunits is disrupted in these assays, which may influence binding affinity determination [149]. 

Functional HTS, fluorescent-cell based assays are advantageous, because they can address some of the problems above mentioned. For example, using heterologous expression systems, the Ca_v_2.2 α subunit can be co-expressed with auxiliary *α2δ* and β subunit to produce biophysical properties more similar to the native channel [124]. In one study, Kir2.3, a potassium channel, was co-expressed together with Ca_v_2.2 α and auxiliary subunits, to control membrane potential [145]. This mechanism allowed identification of novel state-dependent inhibitors [145]. In addition, cell lines expressing Ca_v_2.2 and auxiliary subunits endogenously can be used in HTS to provide information on the mechanisms of novel toxins inhibition, in a native context [149]. Thus, functional HTS assays are expected to accelerate identification, pharmacological characterization and selectivity profile of novel Ca_v_2.2 modulators.

## 5. Conclusions

Animal venoms are rich sources of Ca_v_ channel modulators. Cone snail venoms in particular have provided a diverse array of inhibitors [9], including the most subtype-selective Ca_v_2.2 inhibitors known [8]. These peptide toxins are valuable drug leads, pharmacological tools and drugs in their own right [9,115,117]. In addition, Ca_v_2.2 inhibitor toxins have served as templates for the development of peptidomimetic small molecules, which can then be engineered in an attempt to circumvent some of the disadvantages inherent to peptidic Ca_v_2.2 inhibitors, especially the need for intrathecal use. Key strategies for improving the therapeutic potential of calcium channel include identification of inhibitors of Ca_v_2.2 splice variants that are only expressed in pain states [28], inhibition of Ca_v_2.2 in combination with specific auxiliary subunits [35], optimizing state and/or use-dependent inhibition, and targeting other Ca_v_2.2 regulatory pathways [148]. 
